# Editorial: Photobiomodulation therapy for brain disorders

**DOI:** 10.3389/fneur.2024.1495715

**Published:** 2024-09-26

**Authors:** Chongyun Wu, Tao Wu, Luodan Yang

**Affiliations:** Laboratory of Exercise and Neurobiology, School of Physical Education and Sports Science, South China Normal University, Guangzhou, China

**Keywords:** photobiomodulation, brain disorders, low-level light (laser) therapy, Alzheimer's disease, neurodegeneration

## Introduction

Photobiomodulation (PBM), previously termed low-level light (laser) therapy (LLLT), has been receiving tremendous attention in the prevention and treatment of various brain disorders, including neurodegenerative diseases, brain injuries, and psychiatric disorders (1, 2, 5, 6). PBM therapy, a light-based, non-invasive approach, involves the application of low levels of visible light with a wavelength between 400 and 720 nm or near-infrared light with a wavelength around 700–1,100 nm on biological tissues to regulate biological effects. A large body of research shows that cytochrome c oxidase (CCO) in the mitochondria has been the primary target of PBM. PBM could enhance mitochondrial function by promoting nitric oxide (NO) photodissociation from CCO, thereby increasing CCO activity. However, in recent years, there has been growing debate over whether CCO is indeed the primary target of PBM (3, 4). Additionally, many questions regarding the application of PBM in the treatment of brain disorders remain to be addressed, including light source parameters, irradiation dosage, treatment location, optimal irradiation protocols, and clinical translation. This Research Topic aims to collect studies on the latest advances in PBM modulation on brain disorders, summarizing the most recent findings and uncovering novel discoveries regarding the biological mechanisms of PBM. We aim to highlight the promising therapeutic potential in various brain disorders, its protective mechanisms, laser application treatment regimens, and its clinical translational significance. This Research Topic includes four publications: one clinical trial, one study protocol, one policy and practice review, and one review article. These articles provide valuable insights and practical approaches for applying PBM therapy in the treatment of various brain disorders and emphasize the importance of optimizing PBM parameters, such as light penetration, energy delivered, and delivery methods, to maximize its efficacy.

## Light/laser source parameters shaping PBM outcomes

Although increasing evidence supports the neuroprotective effects of PBM in various brain disorders, the beneficial effects of PBM depend on several illumination parameters, including wavelength, light penetration, plus frequency, power density, energy delivered, scatter, refraction, and coherence. However, the disagreement on light/laser source parameters significantly affects the translation from animal research findings to clinical applications. Among these parameters, the penetration of the light/laser source is an essential factor influencing the photonic energy delivered to the targeted tissue. In a policy and practice review of our current Research Topic, Henderson emphasizes that although emerging commercial products and studies claim therapeutic benefits to patients with low-power devices, the light cannot adequately penetrate the human scalp and skull. In this review, Henderson examines the penetration of infrared light in the treatment of human brain disorders and discusses the penetration of infrared light through specific tissues and heterogeneous tissues in transcranial PBM. In addition, this review suggests that PBM, when applied using a low-power infrared device that doesn't illuminate the target neurons with sufficient fluence, may still exert beneficial effects through systemic mechanisms rather than direct action on neuronal mitochondria in brain disorders.

Following Henderson's review, it's evident that the successful application of PBM depends on the careful design of clinical studies, which need to consider various illumination parameters. With this regard, another contribution to our Research Topic provides valuable insight into the application of PBM in Alzheimer's disease (AD) patients. In this protocol, Yokoi et al. outline a well-structured approach to assessing the efficacy and safety of PBM in this population. The trial design involves a single-center, parallel-group, randomized, sham-controlled study, where AD patients receive either sham or active stimulation for 20 min per session for 12 weeks. The stimulation is delivered using invisible near-infrared light through a combination of applicators placed in the nostril and scalp, targeting both frontal and occipital regions. By measuring outcomes such as cognitive function and caregiver burden, this protocol will provide critical data on the potential therapeutic benefits of PBM in AD, addressing both the efficacy of the treatment and its practicality in a home setting.

In addition, in line with the importance of designing effective PBM interventions, another contribution to our Research Topic further explores AD pathophysiology, factors affecting AD pathogenesis, the role of PBM in modulating AD pathophysiology, clinical evidence and limitations of PBM on PBM therapy for AD, and future direction. Lim claimed that PBM can address multiple aspects of AD pathophysiology by improving mitochondrial function, reducing oxidative stress, and enhancing cerebral blood flow. Importantly, PBM's non-invasive nature and safety make it a promising approach for AD treatment, though further large-scale clinical trials are essential to validate these findings. Moreover, this review highlights the vital role of light/laser source parameters in shaping PBM outcomes and suggests that electroencephalography (EEG) helps optimize PBM parameters, which can significantly influence outcomes. EEG feedback could help personalize PBM treatment, offering a path to more precise interventions aimed at halting AD progression. In this context, integrating artificial intelligence could further refine PBM therapies, ensuring they are tailored to each patient's unique neurological profile. Moreover, a clinical trial article conducted by Miyahara et al. on our Research Topic investigated the efficacy of laser cane therapy on Parkinson's disease, wherein the visual cures rapidly access the motor cortex and are applied to increase visual reliance and alleviate proprioceptive impairment. Although this clinical trial is not directly related to photobiomodulation, it expands the application of laser therapy in brain disorders.

## Epilog

Together, these articles demonstrate the potential of PBM or laser therapy as a multifaceted therapeutic strategy in AD, encouraging future research to optimize parameters and study the long-term effects of PBM therapy. Moreover, more studies are still needed to uncover the novel underlying mechanism of how PBM modulates neural and immune responses, enhances neuroprotection, and promotes neuroplasticity, which can offer a comprehensive view of PBM's therapeutic potential.
